# ECCParaCorp: a cross-lingual parallel corpus towards cancer education, dissemination and application

**DOI:** 10.1186/s12911-020-1116-1

**Published:** 2020-07-09

**Authors:** Hetong Ma, Feihong Yang, Jiansong Ren, Ni Li, Min Dai, Xuwen Wang, An Fang, Jiao Li, Qing Qian, Jie He

**Affiliations:** 1 Institute of Medical Information/Library, Chinese Academy of Medical Sciences and Peking Union Medical College, Beijing, China; 2 Office of Cancer Screening, National Cancer Center/National Clinical Research Center for Cancer/Cancer Hospital, Chinese Academy of Medical Sciences and Peking Union Medical College, Beijing, 100021 China; 3 Department of Thoracic Surgery, National Cancer Center/National Clinical Research Center for Cancer/Cancer Hospital, Chinese Academy of Medical Sciences and Peking Union Medical College, Beijing, China

**Keywords:** Medical parallel corpus, Cancer information, Cross language, Bilingual, Cancer data, HealthNLP

## Abstract

**Background:**

The increasing global cancer incidence corresponds to serious health impact in countries worldwide. Knowledge-powered health system in different languages would enhance clinicians’ healthcare practice, patients’ health management and public health literacy. High-quality corpus containing cancer information is the necessary foundation of cancer education. Massive non-structural information resources exist in clinical narratives, electronic health records (EHR) etc. They can only be used for training AI models after being transformed into structured corpus. However, the scarcity of multilingual cancer corpus limits the intelligent processing, such as machine translation in medical scenarios. Thus, we created the cancer specific cross-lingual corpus and open it to the public for academic use.

**Methods:**

Aiming to build an English-Chinese cancer parallel corpus, we developed a workflow of seven steps including data retrieval, data parsing, data processing, corpus implementation, assessment verification, corpus release, and application. We applied the workflow to a cross-lingual, comprehensive and authoritative cancer information resource, PDQ (Physician Data Query). We constructed, validated and released the parallel corpus named as ECCParaCorp, made it openly accessible online.

**Results:**

The proposed English-Chinese Cancer Parallel Corpus (ECCParaCorp) consists of 6685 aligned text pairs in Xml, Excel, Csv format, containing 5190 sentence pairs, 1083 phrase pairs and 412 word pairs, which involved information of 6 cancers including breast cancer, liver cancer, lung cancer, esophageal cancer, colorectal cancer, and stomach cancer, and 3 cancer themes containing cancer prevention, screening, and treatment. All data in the parallel corpus are online, available for users to browse and download (http://www.phoc.org.cn/ECCParaCorp/).

**Conclusions:**

ECCParaCorp is a parallel corpus focused on cancer in a cross-lingual form, which is openly accessible. It would make up the imbalance of scarce multilingual corpus resources, bridge the gap between human readable information and machine understanding data resources, and would contribute to intelligent technology application as a preparatory data foundation e.g. *cancer-related machine translation*, *cancer system development towards medical education, and disease-oriented knowledge extraction*.

## Background

Cancer is “a generic term for a large group of diseases that can affect any part of the body”, also known as tumor and neoplasm, which takes a large proportion of responsibility for death around the world [1]. In the United States, cancer is the top 2 leading cause for death, and according to statistics, 1,762,450 new cancer cases and 606,880 cancer deaths are predicted in 2019 [2]. In China, there are 4,285,033 cancer cases in 2018 and 2,865,174 corresponding death cases in the same year [3]. According to Globocan from International Agency for Research on Cancer(IARC), the total number of cases in cancer was up to 18,078,957, and 9,555,027 death cases were found in 2018 [4].

All the data evidence show that cancer is an unneglectable, destructive disease for human health which should not be ignored. While the patients and the public should understand cancer better to prevent it, medical experts and scientists also do their research on it for valuable discovery. Thus, dissemination, education and mining of cancer information via different countries will benefit as large scale as possible in a global view. Compared with manual processing methods, the massive medical data is more suitable for developing data-driven algorithms and models. Advanced intelligent techniques e.g. *neural network, natural language processing (NLP),* etc. have been developed to work on this specific hotspot. The intelligence application of NLP method on health especially on cancer, which could both improve the physician perspectives and share cutting-edge science performance to the public, is a great choice for better health research. To achieve this, the data foundation on a specific theme should be prepared first. This leads to the importance of collecting most updating research or information recorded in various languages.

All sorts of resource related, such as electronic health records (EHRs), medical knowledge on a specific disease, other information from institutions or hospitals; the cognition and knowledge capacity of physicians, professionals, and other medical staffs etc. contain large amount of cancer information. However, most of them are non-structural text that cannot be directly used for machine learning. They need to be transformed into the structural data for information extraction and network training. On the other hand, imbalanced scale of data resources in different languages may result in knowledge distribution disparity [5]. In addition, cross-lingual language processing requirements have been desired by most medical practitioners, who aim at obtaining more health-related findings, better ways to utilize data automatically and finally improving health quality. The same request is raised by data scientists of medical informatics. Therefore, cross-lingual resources towards medical education, knowledge dissemination, and intelligence application play an important role in promoting knowledge diffusion and exchange.

Many efforts towards cross-lingual knowledge dissemination have been made from expert translators, machine translation, and crowd-sourcing (e.g. *Wikipedia* [6]). Compared to costly, time-consuming manual translation, automatic machine translation is more popular, which is supposed to be humanlike and correct despite multilingual similarities and ambiguities [7]. MulTed is a multilingual parallel corpus collected from TED talks containing general topics [8] etc. Still, the medical field shows its specialty, which brings more challenges and requires a higher quality of translation where the high-quality parallel corpus is needed [9]. Few have been provided in medical domain, for example, the Mantra GSC was a multilingual corpus developed for biomedical concept recognition focused on terminology [10]. But when it comes to the cancer domain, the lack of corpus resources is obviously shown.

Considering the requirements for paramount disease education and dissemination, descriptive multilingual knowledge base construction, and cross-lingual natural language processing technique implementation, we found that the best way to fulfill all the demands is to create a cross-lingual parallel corpus in cancer information first. A parallel corpus is a document collection composed of two or more disjoint subsets, each written in a different language as the translation to the others. A good parallel corpus has a high request for content, either in a specific realm or aligned pattern. The ideal cancer parallel corpus should be authoritative, comprehensive, systematic, and cross-lingual, also covering substantial information for human health such as treatment, prevention, risk factors, genetics, characteristics, etc.

Constructing a cross-lingual cancer parallel corpus is nontrivial and is challenging in the reliability, alignment precision and information abundance. It could bring a better training performance, a higher utilization, and a better knowledge dissemination via applications such as machine translation.

### Related work

#### Workflow

Many research built their own corpus and provided construction workflow as references. Dalianis et.al constructs a parallel corpus for creating a bilingual dictionary with word alignment methodology [11]. The ICTCLAS tool is used for word segmentation, and the sentence alignment is performed by 60 h manual work, while the evaluation also came from the manual process [11]. It provided possible routes on sentence alignment, word segmentation, and corpus evaluation.

Koehn raised a workflow on parallel corpus construction, which could be summarized as obtaining raw data, document alignment, sentence splitting, normalization, tokenization, and sentence alignment. The corpora in different languages were stored separately [12]. Chen and Ge succeeded establishing a parallel corpus of medical works based on python, where the corpus construction workflow was text collection, text preparation & processing including text scanning, error recognition, proofreading, source and translation texts separation, and automatic sentence alignment [13].

Rafalovich and Dale constructed a six-language parallel corpus using United Nations general assembly resolutions encoded in XML, which showed the corpus analysis process including content, title, the number of perambulatory and other researches [14]. The main component in the table was number which led to stripping tables as the only table-process pattern while we extract context from our corpus tables and align them.

In a parallel corpus of Balkan language construction, south-east times were used as the data source, and construction workflow was designed as data preparation using web spider, data collection according to UTF-8, sentence splitting, sentence alignment, and test set [15]. Another corpus consisted of TED talks was designed in a similar but simpler workflow, which is crawling data with original HTML format, and align them according to specific strategies [16]. In addition, when it comes to multilingual parallel knowledge graph construction, data processing, cross-lingual knowledge graph building, data quality improvement, and application were designed as the workflow [5].

#### Alignment

Sentence alignment is a key component in parallel corpus construction. In summary, there are three categories in automatic sentence alignment, respectively statistical methods on numbers and sizes, linguistic methods on linguistic information and hybrid methods involving both [17].

Linguistic methods are not always independent, but to be combined with statistical ones, making it the hybrid method with better performance. One of the most typical methods was Gale and Church’s, where forehead paragraphs alignments or anchor points were needed [18]. Moore’s was the combination of sentence length and statistical probability [19]. Chuang and Yeh’s method made use of the punctuation marks in order to align sentences [20]. Wu’s method was based on length to align the English-Chinese corpus [21]. Hunalign method inserted a bilingual dictionary to obtain better accuracy [22]. Bluealign completed the target text alignment with the machine translation of source text instead of direct alignment [23].

## Methods

### Data source

The selection of parallel corpus shall be done carefully with strict criterion. We choose Physician Data Query (PDQ) as the original corpora, which is the U.S. NCI’s (National Cancer Institute) comprehensive source of cancer information, and also the knowledge base carrying weekly updating. NCI encourages knowledge spreading and cancer education in different languages. PDQ in other language versions such as Chinese is provided for extended reading. Compared to other cancer knowledge base or information systems, PDQ is more competent for its systematicness, authoritativeness, and comprehensiveness in cancer.

**Fig. 1:**
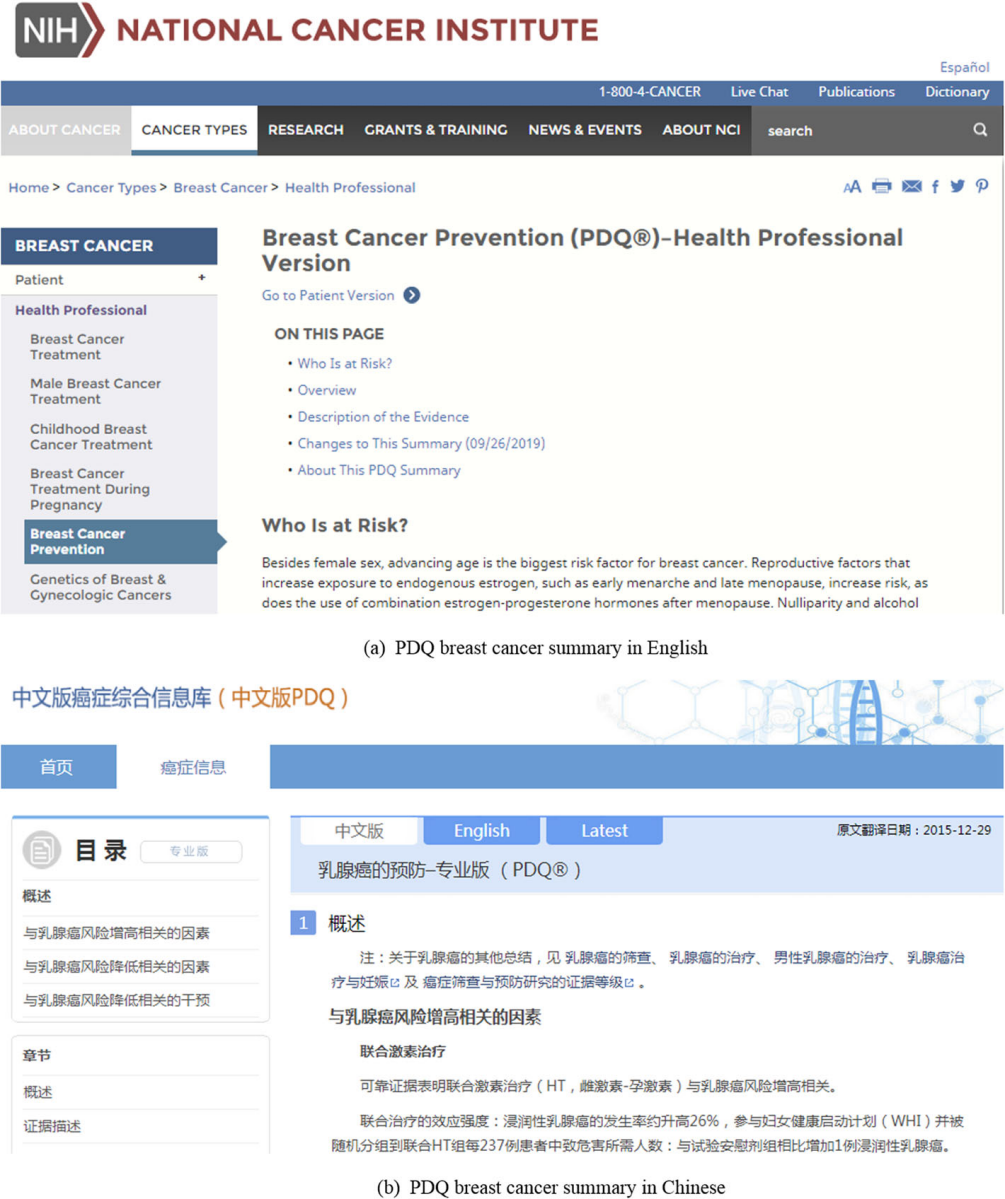
Example webpages of PDQ_EN_ and PDQ_CH_

The Chinese version of PDQ (PDQ_CH_) was rigorously translated by experienced clinicians and experts who performed proficient skills in both languages and worked in a recognized cancer hospital in China. With the quality control of two top cancer institutes in the United States and China, the dataset was considered as the golden standard. The original data format was XML, and it described the information of a wide range including cancer, drug, genetic term, and other related information [24]. Figure 1 showed the user interface among the entire knowledge base which is open to the public, i.e. Figure 1 Example webpages of PDQ summaries in English and Chinese [25].

The original corpus consisted of six cancer types, respectively liver cancer, colorectal cancer, breast cancer, lung cancer, stomach cancer, and esophageal cancer. Three themes were contained inside cancer types including cancer prevention, cancer screening, and cancer treatment. There were 22 pairs of XML files in total. By analyzing the raw data which is full of embedded elements, we found the number of English sentences, Chinese sentences, English words, and Chinese words were respectively 22,091, 12,365, 291,095, and 170,688 [9].

### Corpus construction workflow

We improved our workflow according to the referred work. Most of them were in general domain, few in medical domain without a particular subject while our corpus only focused on cancer. There were multiple linguistic granularity of text alignment, including paragraph, sentence and word, which could be extracted for different purposes. We decided to use XML format for corpus, which was easier to operate and more efficient.

**Fig. 2:**
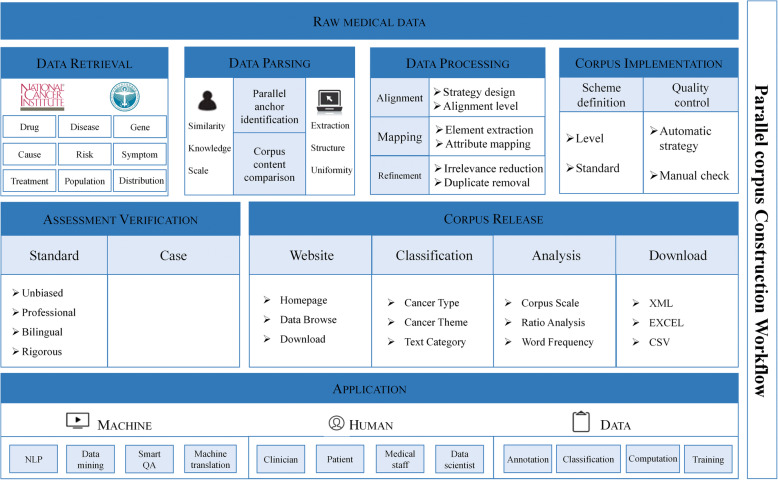
The Parallel Corpus Construction Workflow

Following the framework shown in Fig. 2, our construction workflow included 4 elements, respectively data, technique, assessment, and application. It could be specified into 7 stages: (1) Data Retrieval, where we collected the data from the National Cancer Institute (NCI) and Cancer Institute, Chinese Academy of Medical Sciences (2) Data parsing, where we identified parallel anchor and compared both corpora. (3) Data processing, where we designed alignment strategy and implemented data mapping (4) Corpus implementation, where we defined corpus construction schema and completed corpus quality control (5) Assessment verification, where we determined the evaluation.

criteria and assessment criteria (6) Corpus release, where we built corpus website for public visiting, which showed the classification and analysis of parallel corpus (7) Application, where we listed for whom and for what scenarios our corpus could be reused.

There were two main problems when trying to make the corpus parallel. First, despite PDQ_CH_ came from the manual translation of PDQ_EN_, layers, elements inside XML and even the whole structure were not always the same [9]. Specifically, it was common that one sentence in PDQ_EN_ responded to none information in PDQ_CH_. Some data elements could not be found. We assumed there might be several possible reasons. Translators were experts in cancer, but few of them were professionals in data. This may lead to a misoperation during data processing. Also, full-time data processors could put the wrong translated information due to the lack of clinical knowledge and English proficiency. The other great difficulty came from the difference of element occurrence, i.e. *frequency of the same elements* [9].

Data retrieval is described in the above data source session. Data parsing, data processing, corpus implementation, and assessment will be introduced in the following session. Corpus release and application is illustrated in the results and discussion session.

### Data parsing

To solve the two problems mentioned above, we analyzed and compared both corpora first. The purpose of this stage was corpus content comparison and parallel anchor identification. As was edited in XML format, it showed many elements containing a mass of sentence pairs and paragraph pairs. These text pairs were the main content we aimed at due to the abundant core information. Element was a data component in XML structure, in this case, the original data were consisted of different elements with texts inside. We compared every element in two corpora and listed the shared and different ones. The shared ones existing in both corpora would be extracted, those only appeared in one corpus were excluded because no correspondence in their counterpart corpus could be found so that none parallel content would exist.

After counting the occurrences of shared elements in both corpora, we found that most elements were not completely accordant. We analyzed all the uniformity and difference of elements, discovering that 38 of 54 elements were identical in both corpora. Thus, we selected the shared 38 elements for parallel corpus construction.

As for the content alignment problem, alignment anchors were needed. Through the analysis in data structure, we discovered one element - the ‘para’ element, with the meaning of a paragraph, had an ‘ID’ that can help to locate and to align. ‘Para ID’ was the unique identifier that marked a paragraph so that it could be easily found. ‘Para’ element was the only element appeared exactly same times in both corpora, so it was the best choice that we could select for alignment. Same ID could help locating the same content. The other elements either had none ‘ID’ for locating, or they did not occur same times in both corpora, which brought great difficulty ahead of alignment. We listed the element ‘Para’s and their affiliated paragraphs, which were the main parallel corpus composition.

### Data processing

The whole process in this stage was alignment, mapping and refinement. We needed to complete the strategy design and determine alignment level. Element extraction and attribute mapping inside elements would then be fulfilled for mapping. At last, in the refinement part, we eliminated the irrelevant information and kept the meaningful ones, and removed the duplicated content.

Most alignments in other research were parallel in sentence level, our corpus contains hybrid alignment, i.e. parallel *words, phrases, and sentences*. Based on the particular corpus structure, we designed our own improved alignment strategy to ensure accuracy. According to the default parallel anchor ‘Para ID’, we automatically aligned all the corpus elements through the sequence. Each other element was supposed to be in the middle of two ‘Para IDs’. In this way, we found some differences in the number of elements, we manually checked and aligned them. The proportion of the unaligned part was relatively small (1.83%). After element alignment, the text was extracted to get prepared for fine-grained alignment.

For aligned paragraphs, we started sentence splitting to compare sentence numbers in two corpora so that we could comprehend the proportion of sentence pairs with one-to-one correspondence and one-to-many correspondence. In the meantime, we collected as many abbreviations, decimals and other situations as possible to eliminate their influence in sentence splitting, e.g. without settled rules for this abbreviation, U.S. could be split with U as the end of former sentence and S as the beginning of the latter sentence. Sentences were automatically aligned in this part according to delimiters and their sequences, and a small part not aligned were manually checked and adjusted. For the other element ‘table’ in the corpus, there were many conditions with numbers-only, letters-only context, yet it was meaningless. We extracted the meaningful content and removed the duplicated ones including inner files and cross files. In this way, we could simplify the corpus and present more valuable text with less noise.

### Corpus implementation & assessment

To complete the construction, the corpus schema was designed as follows: all text should be classified into words, phrases, and sentences. Words were mostly subtitles from the original corpus and could support terminology dictionary construction etc. Phrases were not identical with sentences, the former had shorter word counts and may present a generality for the following paragraphs. The rule was set by length, those with 1–2 words were sorted into ‘Word’, those with 3–7 words were classified into ‘Phrase’, and those over 7 words were considered as ‘Sentence’.

To evaluate the alignment performance, a strategy must be designed. The precision of both medical knowledge and alignment must come first in the medical area, otherwise, misunderstanding and ambiguity might lead to deviation. For the same reason, we came up with four standards for evaluator selection in the assessment, namely unbiased, professional, bilingual and rigorous. Two evaluators should be participated to avoid bias, conduct a discussion, and a third party was initialized in disagreement. Meanwhile, professionals both in medicine and information, basically medical informatics, were invited to make decisions. Misunderstanding in English or Chinese might bring a negative impact on the assessment result, so we chose experts with good education experience from overseas in the first place. Considering cancer information as a strict science, we put ‘rigorous’ as a must-be standard and selected researchers for evaluation.

The criteria can be categorized into five types: aligned & correct, aligned & partial correct, aligned & incorrect, partial aligned, unaligned. To guarantee the corpus precision, we deleted those not aligned or correct. There were 8 out of 420 word pairs, 9 out of 1092 phrase pairs, and 64 out of 5254 sentence pairs that did not meet the criteria, i.e. not aligned or correct. After constructing our parallel corpus, we build a website for it to be accessible, open sharing and reusable.

## Results

### Corpus overview

We constructed a 22-pair cancer parallel corpus, classified in cancer type, cancer theme, and text type. PDQ_EN_ and PDQ_CH_ were separately stored but combined with the same ID, which was easy for users to operate in the database. The parallel corpus consisted of 6 cancers, 22 detailed subsets, and 6685 pairs including 412 words, 1083 phrases and 5190 sentences. After quality assessment, we found there were five situation: aligned & correct, aligned & partial correct, aligned & incorrect, partial aligned, unaligned. The examples of these five types were shown in Table 1.

**Table 1:** Example content of alignment

Topic	Example of corpus alignment evalution
*Aligned & Correct*	Bevacizumab can reasonably be added to either FOLFIRI or FOLFOX for patients undergoing first-line treatment of metastatic colorectal cancer 贝伐单抗可以合理的加入FOLFIRI或FOLFOX化疗方案, 作为一线方案治疗转移性结直肠癌患者
*Aligned & Partial Correct*	Weight was the strongest predictor, with a RR of 2.85 (95% CI, 1.81–4.49) for women weighing more than 82.2 kg, compared with those weighing less than 58.7 kg 体重是最显著的预测因素, 体重大于82.2kg的女性与体重低于58.6kg的女性相比, RR为2.85(95%CI, 1.81–4.49)
*Aligned& Incorrect*	Because patients with SCLC tend to develop distant metastases, localized forms of treatment, such as surgical resection or radiation therapy, rarely produce long-term survival 生存时间延长的SCLC患者中多数是因为参与临床试验, 得到了最佳、最适合的治疗
*Partial aligned*	The overall 5-year survival rates were 76 and 74% for preoperative and postoperative chemoradiation, respectively (*P* = .80) 术前放化疗组和术后放化疗组的5年总生存率分别为76%和74%(*P* = 0.80)、5年累积局部复发率分别为6%和13%(*P* = 0.006)
*Unaligned*	Lethal toxicity was less than 1%, with grade 3 to 4 hematologic toxicity in 55 and 49% of patients in the two bolus arms, respectively (i.e., arms 1 and 3) versus 4% of patients in the continuous-infusion arm 三组的DFS、OS和局部失败率(LRF)未见差异

**Fig. 3:**
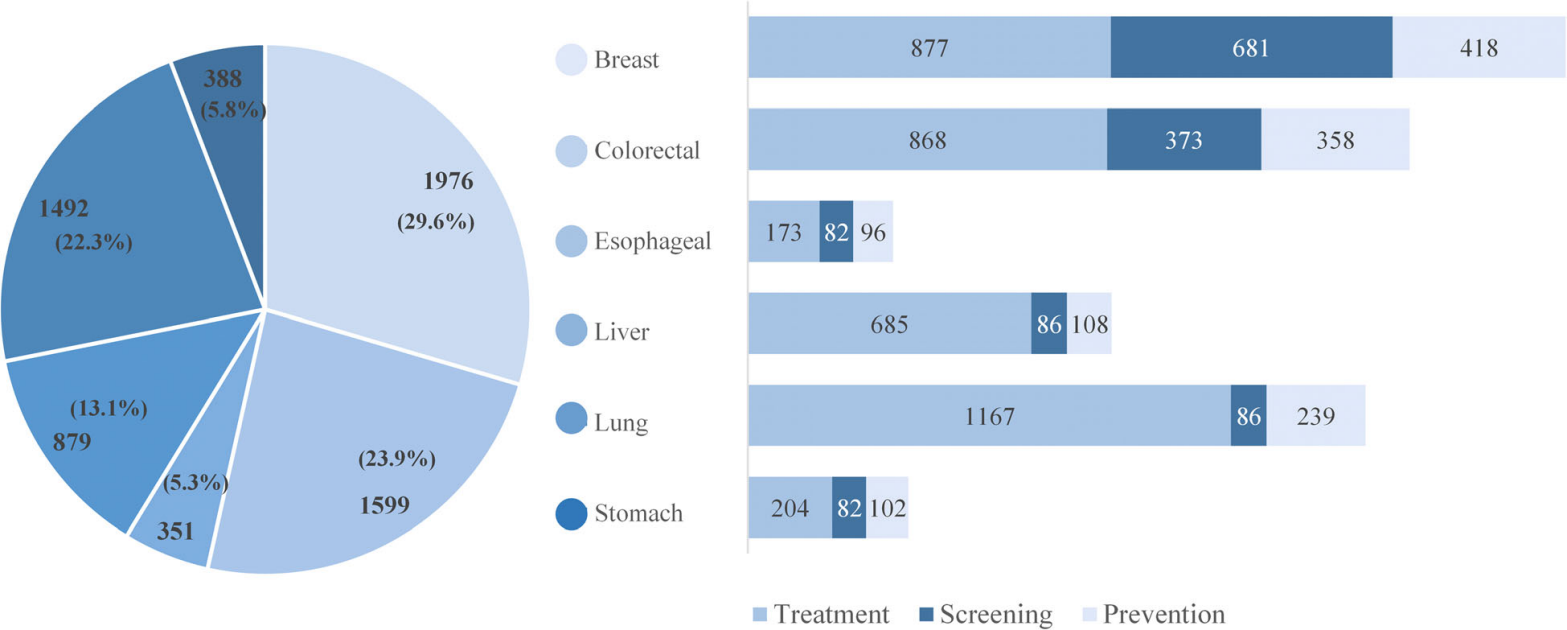
Corpus statistics in different cancers and themes

Apart from the general descriptive corpus, we analyzed the number of different cancer types and texts. Breast cancer, colorectal cancer, and lung cancer took the most of the entire corpus, respectively 30, 24, and 22%. The number of corpus pairs in these three cancer types summed up to more than three quarters, left other cancer types only 24% in total. Among the corpus in all cancer types, the treatment theme accounted for the largest proportion in each cancer type, with the total number as 3974 of 6685 i.e. 59.45% (Fig. 3).

### Corpus analysis

**Fig. 4:**
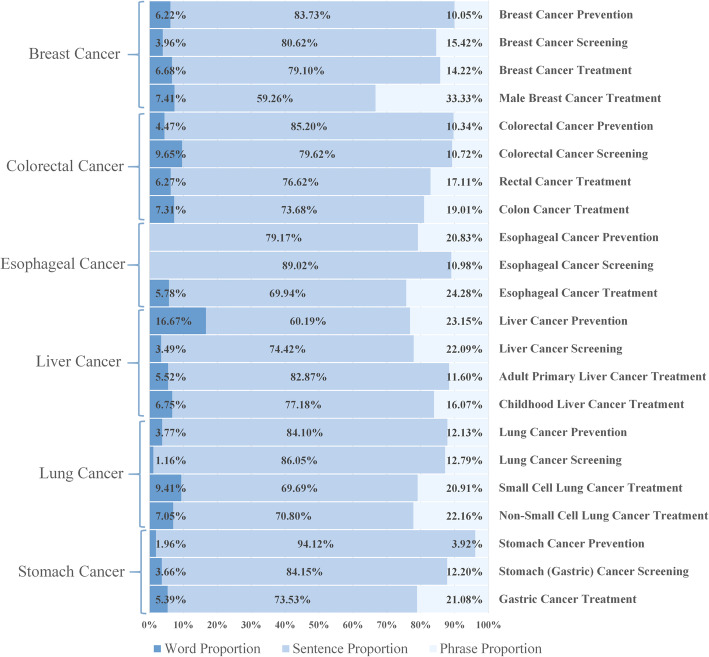
The Proportion of word, phrase, and sentence in different cancer theme

For obtaining the corpus text distribution, we analyzed the proportion statistics in each theme of each cancer type, the results are shown in Fig. 4. We listed each cancer type with prevention, screening, and treatment as a fixed order. Among them, breast cancer treatment could be divided according to sex. Colorectal cancer treatment could be divided into colon cancer and rectal cancer. Liver cancer treatment could be divided into adult primary and child cancer treatment. Lung cancer treatment could be classified by whether they are small cells or not.

There were no word pairs in two cancer themes, both belonged to esophageal cancer. Sentences were the main corpus resource for each file, taking proportion from 59.26 to 94.12%, with an average of 77.86%. Meanwhile, words only accounted for an average 5.6% from 0 to 16.67%. Phrases were mostly subtitles extracted from the original dataset, which took an average ratio of 16.55% and the maximum proportion was 33.33%.

### High-frequency word distribution statistics

We performed word tokenization in this stage and analyzed its high frequency word ranking. ‘Natural Language Toolkit’ [26] was used for English tokenization and ‘jieba’ [27] for Chinese tokenization, both used to obtain word frequency statistics. Higher word frequency indicates more importance. A rule was set for choosing words, those part of speech including adverbs, adjectives, pronouns, numerals, verbs, articles, prepositions, conjunctions were deleted and only meaningful nouns were saved. We ranked the word according to word frequency in descending order.

**Fig. 5:**
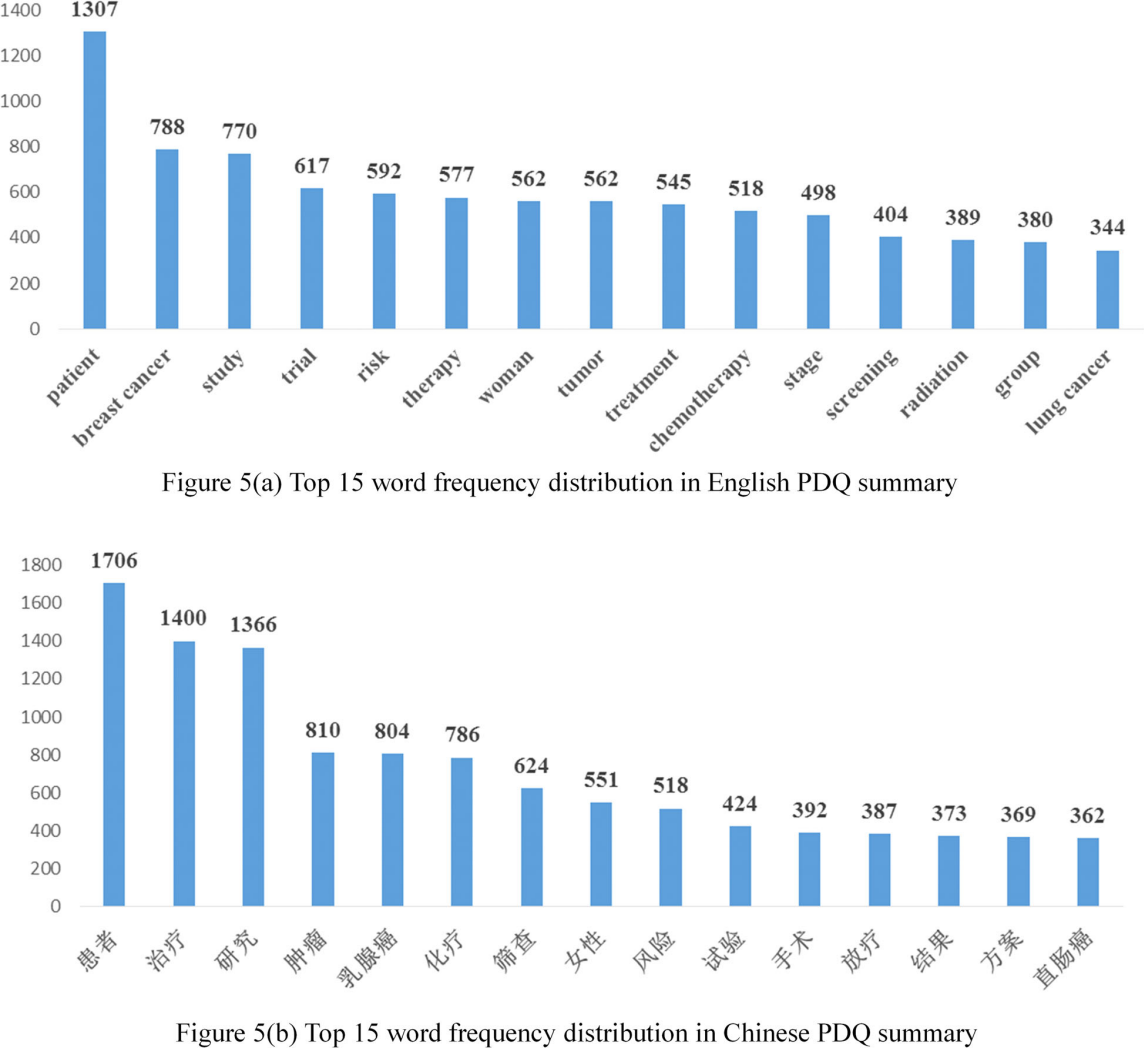
Word frequency distribution of PDQ summary in English and Chinese

We selected top 15 word frequency and showed its distribution among the whole corpus. The results from both corpora were not the same, either in statistics or word itself. The PDQ_EN_ had 11 words perfectly matching to their top 15 counterparts. Both top words mentioned ‘patients’ which ranked double first where we inferred patients might have been put in the central position; women were brought up more, more than 500 times, showing that women might account for a large part in cancer patients or in great risk getting one; trial, risk, treatment, chemotherapy, radiation were also important stages in cancer cycle also mentioned in top word frequency. The different word frequency may result from different translation to the same word. The word “治疗” appeared 1400 times in PDQ_CH_, while in PDQ_EN_ there were several words corresponding e.g. therapy and treatment etc. The other reason possibly responsible for this may due to the tokenization of conventional expression, e.g. the word “stage” in PDQ_EN_ usually occurred with roman numerals representing the cancer stage. However, in many cases, word tokenization would not split the corresponding Chinese translation “期” out. In addition, some words would appeared with an invisible or supplemental translation for better fluency, e.g. the word “方案” in Chinese could be aligned with “regimen”, “option” in English, and could also not occurred in the original English corpus, e.g. the translation of sentence “Drug combinations described in this section include the following” was “本章节将介绍下列化疗联合用药方案”. All of these above linguistic phenomenon represented the adaptation to different languages. Figure 5 showed the word frequency of cancer summary in different languages and its statistics.

### Corpus release & access

**Fig. 6:**
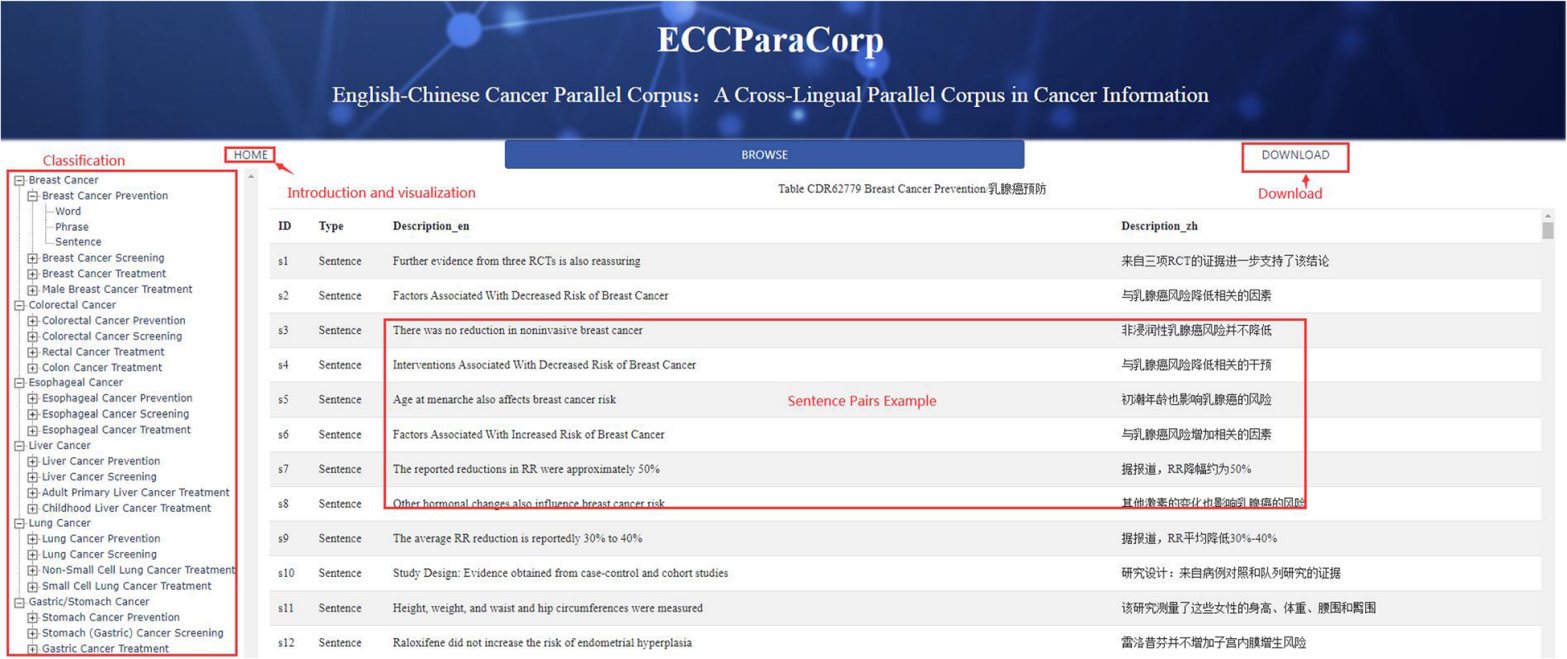
The user interface of ECCParaCorp

**Fig. 7:**
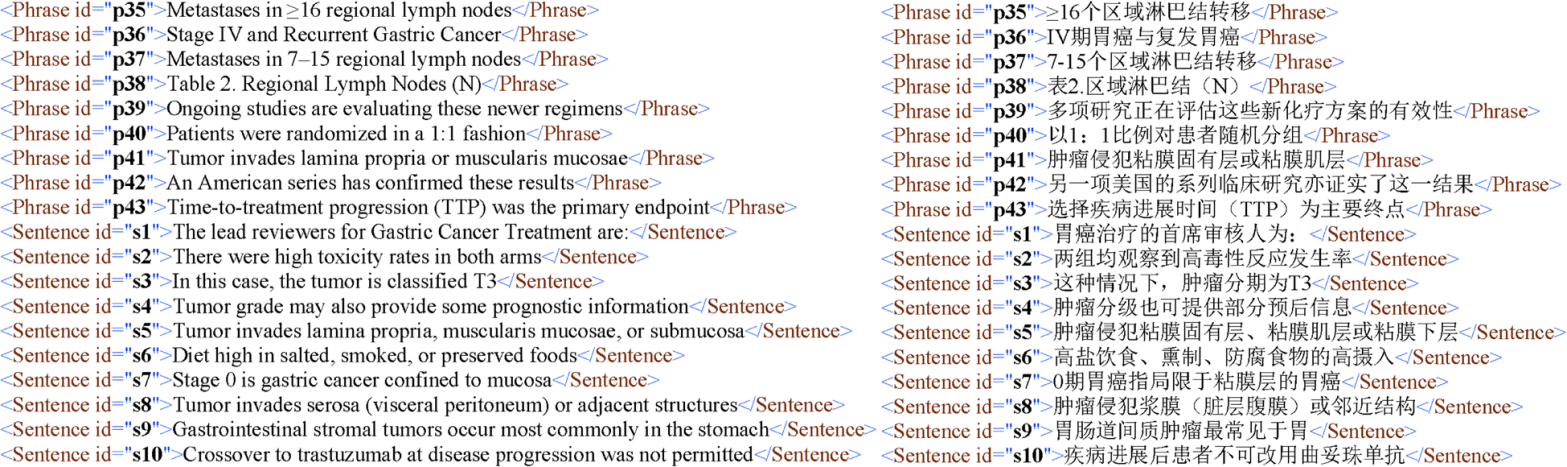
Corpus in XML format

To promote the usability and deep mining of our parallel corpus, we built a user-friendly interface with all data accessible for users (Fig. 6). In the home page, users could see the introduction and relative statistics, charts, and diagrams visualizing the corpus. In the browse page, users could browse the corpus demonstration classification on the left bar, specifically 6 cancer types including liver cancer, colorectal cancer, breast cancer, lung cancer, stomach cancer, and esophageal cancer. They each had subset in prevention, screening and treatment theme, below which showed different classified text type involving word, phrase, and sentence. Users could see our data organization structure and freely chose the subject they were interested in. In the download page, users could download the cancer parallel corpus in three formats, respectively XML (Fig. 7 shows an example), CSV and EXCEL, each with their function and characteristic for different data processing advantages and purposes. The part download is also available.

## Discussion

### Principle finding

Health improving is getting more and more attention, and cancer is of high concerns among all diseases. The requirement for cancer information is not only raised by professionals and the general public in different countries who cared about their health, but artificial intelligence methodology e.g. *modeling, algorithm* as well. An openly accessible cancer-related parallel corpus is a prerequisite preparation and great beginning for multiple dimension analysis. Therefore, we developed the ECCParaCorp which illustrated the most reliable cancer information in different languages from the most authorized institutes. ECCParaCorp currently consists of 6 cancer types, 3 cancer themes, 22 subsets. The possible applications will be discussed below.

### Corpus peculiarity

Compared to other studies, our corpus is preferable in the following sections: (1) Data reliability. Published from National Cancer Institute, the Physician Data Query cancer information converges authoritative and comprehensive evidence-based cancer information summaries specifically for health professionals. (2) Cancer focus. Different from other general medical corpora, ECCParaCorp only focused on cancer, which is deeper and more specific. As far as we know, no similar parallel corpus focused in cancer has been established. (3) Multi-dimensional application. Cancer parallel corpus could be used in knowledge dissemination through various technique e.g. *neural network training for machine translation, knowledge computation for knowledge graph construction*, and through different pipelines, e.g. *medical education, physician training*.

### Corpus application

With cancer information summary as the main content, this parallel corpus was constructed to provide necessity as the prerequisite resource data for real medical needs. It could bring benefit from many perspectives, e.g. *human, machine, data*, etc.

### Human perspective

There are numerous roles in this perspective. As for clinicians, it is a basic demand to obtain cutting-edge information and look up to evidence e.g. *what is the most updated cancer drug, what are the side effects, how many cases have been proven the efficiency of the drug, what are the components of a drug, is that appropriate for the specific patient?* The evidence-based way has always been advocated. Cancer corpus could efficiently assist them with decision making in terms of evidence. As for medical researchers, they require the corpus to complete more training or mining to access potential value and apply it to more clinical practice. Total different conclusions could be drawn when analyzing from diverse views, e.g. *physicians with clinical expertise and informatics experts with data science expertise*. As for patients, parallel corpus could be their knowledge base to learn more information at a faster pace. Families also urge to acquire more fundamental information and specific knowledge, so that high-quality care can be given. The general public, meanwhile, is also eager to know more about cancer, especially how to prevent it, how to know if someone is highly possible getting it.

### Machine perspective

Data mining could help with value discovery, which is mostly done by machines with rules or parameters set in the first place. As a language resource, our corpus could be the basis where Natural Language Processing (NLP) technique act on. The core concept is to realize human-computer interaction (HCI) through language data processing. With the development of artificial intelligence, HCI will be the interface filling gap between human and real-world data. Other data mining tools could also be used on the corpus for algorithm comparison, modeling prediction, and additional functions. As a descriptive resource, our corpus could be a fair one for building smart questions and answering systems through auxiliary machine reading, understanding, explanation, and generation. It could also be a complementary data source or cancer dictionary, an external forces for improving classification precision, terminology accuracy, and extending validation set. As a bilingual resource, parallel data could be upstage support of cross-lingual automatic translation, i.e. *machine translation*. The importance of real-time recording and dissemination in other languages is self-evident, especially in a case where frontier discoveries were carried by English. The parallel corpus would strongly promote this situation.

### Data perspective

As for data itself, the amount of information it can carry and present is much more. A great amount of preparatory work could be done so that we can understand the data from multiple dimensions. The part-of-speech tagging could help to discover hidden linguistic characteristics. The combination of linguistic characteristics and word embedding shows a potential that more latent cancer features might be found. Semantic annotation could help to realize entity recognition and relationship extraction, increasing the classification veracity for neural network training and boosting training performance. As an educational dataset, the cancer-subject dictionary could be built for specific groups in need. Knowledge computation could be performed on the corpus to explore higher-level nonrepresentational meaning. In brief, there are many meaningful and interesting points of penetration that could work. Table 2 shows an example case of the parallel corpus.

**Table 2:** Example content of parallel corpus

Topic	Example of corpus pairs
*Breast Cancer*	Carriers with a history of breast cancer have an increased risk of contralateral disease that may be as great as 5% per year 有乳腺癌病史且存在BRCA1和BRCA2基因突变的女性同侧复发乳腺癌的风险每年增加5%
*Liver Cancer*	Patients with primary hepatoblastomas that remain unresectable, defined as tumors with less than 1 cm radiographic venous margins, POSTTEXT 3 multifocal or POSTTEXT 4, will be referred to a liver transplant center after the first two cycles of C5VD 对于患有原发性肝母细胞瘤的患者, 若放射影像学显示其距静脉边缘不足1cm、POSTTEXT3期多灶性或POSTTEXT4期肿瘤, 则无法切除。应考虑行2程C5VD后转入肝移植中心
*Lung Cancer*	The lung component of PLCO addressed the question of whether annual single-view (posterior-anterior) chest x-ray was capable of reducing lung cancer mortality as compared with usual medical care PLCO中肺癌筛查部分解答了每年一次单一体位(后前位)胸片较常规医疗相比是否能够降低肺癌死亡率的问题
*Colorectal Cancer*	NSAIDs reduce the risk of adenomas, but the extent to which this translates into a reduction of CRC is uncertain 非甾体类抗炎药可降低腺瘤的发病风险, 但能否表明降低结直肠癌的发病率尚不明确
*Esophageal Cancer*	Patients whose duodenal ulcers were treated successfully with antibiotics developed reflux esophagitis twice as often as those in whom infection persisted 十二指肠溃疡患者使用抗生素完全治愈后发生反流性食管炎的几率是感染持续存在患者的2倍
*Stomach Cancer*	Screening would result in uncommon but serious side effects associated with endoscopy, which may include perforation, cardiopulmonary events, aspiration pneumonia, and bleeding requiring hospitalization 胃癌筛查可能引起罕见、但一旦发生即很严重的内镜相关副作用, 包括穿孔、心肺事件、吸入性肺炎与需要住院的出血事件

### Limitations and future studies

Currently, there are only 6 cancer types collected in the corpus, which has not yet been a comprehensive and systematically integrated cancer knowledge base. More data processing e.g. *annotation* should be done for machine learning. More information extraction techniques could be used for obtaining semantically useful information in the future. There is much work to do in extending the corpus scale, collecting more meaningful text, and analyzing from diverse perspectives.

## Conclusions

ECCParaCorp is an English-Chinese cancer parallel corpus towards cancer education, utilization, application and is now open to the public, providing download service in three formats, respectively XML, CSV, and EXCEL. It could be applied to many scenarios such as clinical decision support, cancer patient education, human-computer interaction, medical informatics value discovery, etc. To the best of our knowledge, ECCParaCorp is the first specialized English-Chinese cross-lingual parallel corpus focusing on cancer, that contain authorized, scaled, cross-lingual parallel cancer information in English and Chinese. Our parallel corpus would be a solid support and insurance for intelligence-based technique development on cancer-oriental health improving.

## Data Availability

The dataset is publically available via http://www.phoc.org.cn/ECCParaCorp/.

## Abbreviations

NCI: National Cancer Institute
PDQ: Physician Data Query;
NLP: Natural Language Processing
HCI: Human Computer Interaction
XML: Extensible Markup Language
CSV: Comma-Separated Values

## References

[1] Cancer World Health Organization. https://www.who.int/en/news-room/fact-sheets/detail/cancer. Accessed 9 Jan 2020.

[2] Siegel RL, Miller KD, Jemal A (2019). Cancer statistics, 2019. CA Cancer J Clin.

[3] Cancer Statistic in China, 2018 World Health Organization. http://gco.iarc.fr/today/data/factsheets/populations/160-china-fact-sheets.pdf. Accessed 9 Jan 2020.

[4] Cancer Statistics in 2018. http://gco.iarc.fr/today/data/factsheets/cancers/39-All-cancers-fact-sheet.pdf. Accessed 9 Jan 2020.

[5] Jin H, Li C, Zhang J, Hou L, Zhang P (2019). XLORE2: large-scale cross-lingual knowledge graph construction and application.

[6] Wikipedia. https://www.wikipedia.org/. Accessed 9 Jan 2020.

[7] Hirschberg J, Manning CD (2015). Advances in natural language processing. Science.

[8] Zeroual I, Lakhouaja A (2018). MulTed: A multilingual aligned and tagged parallel corpus. Applied Computing and Informatics.

[9] Ma H, Ren J, Wang X, Fang A, Li J, Qian Q (2019). A cross-lingual effort towards managing English-Chinese Cancer education resources. Stud Health Technol Inform.

[10] Kors JA, Clematide S, Akhondi SA, van Mulligen EM, Rebholz-Schuhmann D (2015). A multilingual gold-standard corpus for biomedical concept recognition: the mantra GSC. JAMIA.

[11] Dalianis H, Xing H-c, Zhang X (2010). Creating a reusable English-Chinese parallel Corpus for bilingual dictionary construction. Seventh conference on international language resources and evaluation (LREC’10).

[12] Koehn P. Europarl: A Parallel Corpus for Statistical Machine Translation. tenth Machine Translation Summit: Phuket, AAMT; 2005. p. 79–86.

[13] Chen X, Ge S (2011). The construction of English-Chinese parallel Corpus of medical works based on self-coded Python programs. Procedia Engineering.

[14] Alexandre Rafalovitch RD (2009). United Nations General Assembly Resolutions : a six-language parallel corpus. Machine Translation Summit.

[15] Tyers FM, Alperen MS (2010). South-East European Times: A parallel corpus of Balkan languages.

[16] Federico MCCGM. WIT 3 : Web Inventory of Transcribed and Translated Talks. 16th EAMT Conference: Trento, European Association for Machine Translation; 2012.

[17] Mohamed MZ, Ihalapathirana A, Hameed RA, Pathirennehelage N, Ranathunga S, Jayasena S (2017). Automatic creation of a word aligned Sinhala-Tamil parallel corpus. 2017 Moratuwa engineering research conference (MERCon).

[18] Gale WA, Church KW (1993). A program for aligning sentences in bilingual corpora. Comput Linguist.

[19] Moore RC (2002). Fast and Accurate Sentence Alignment of Bilingual Corpora.

[20] Thomas C. Chuang KCY, editor Aligning Parallel Bilingual Corpora Statistically with Punctuation Criteria. Computational Linguistics and Chinese Language Processing; 2005: The Association for Computational Linguistics and Chinese Language Processing.

[21] Wu D, Aligning a parallel English-Chinese corpus statistically with lexical criteria (1994). Proceedings of the 32nd annual meeting on Association for Computational Linguistics.

[22] Varga D, Halácsy P, Kornai A, Nagy V, Németh L, Trón V (2007). Parallel corpora for medium density languages.

[23] Sennrich R, Volk M (2011). Iterative, MT-based sentence alignment of parallel texts.

[24] Physician Data Query (PDQ) National Cancer Institute. https://www.cancer.gov/publications/pdq. Accessed 9 Jan 2020. 10.1093/jnci/djs231 22517987

[25] Physician Data Query (PDQ) Chinese Version Cancer Institute and Hospital, Chinese Academy of Medical Sciences. http://pdq.cicams.ac.cn/ Accessed 9 Jan 2020.

[26] Natural Language Toolkit. http://www.nltk.org/. Accessed 9 Jan 2020.

[27] Jieba. https://github.com/fxsjy/jieba. Accessed 9 Jan 2020.

